# Comparative performance analysis of hemispherical solar stills using date and olive kernels as heat storage material

**DOI:** 10.1038/s41598-025-87448-z

**Published:** 2025-02-28

**Authors:** Reski Khelifi, Tawfiq Chekifi, Abdelfetah Belaid, Mawloud Guermoui, Abdelaziz Rabehi, Ferkous Khaled, Mabrouk Adouane, Ayman Al-Qattan, Takele Ferede Agajie

**Affiliations:** 1 Unité de Recherche Appliqué en Energies Renouvelables, URAER, Centre de Développement des Energies Renouvelables, , CDER, 47133 Ghardaia, Algeria; 2 Telecommunications and Smart Systems Laboratory, University of ZianeAchour, 17000 Djelfa, Algeria; 3 Materials, Energy Systems Technology and Environment Laboratory, Ghardaia University, Ghardaia, Algeria; 4 Institute for Scientific Research (KISR), Kuwait, Chemical Engineering Department, College of Engineering and Petroleum, Kuwait University, Kuwait, 13060 Kuwait; 5 Department of Electrical and Computer Engineering, Faculty of technology, DebreMarkos University, 269, Debre Markos, Ethiopia

**Keywords:** Hemispherical solar still, Date kernels, Olive kernels, Heat storage materials, Distillation efficiency, Solid Earth sciences, Energy science and technology, Engineering

## Abstract

This study investigates the performance of hemispherical solar stills (HSS) enhanced with date kernels and olive kernels as heat storage materials to improve water distillation efficiency. By utilizing these natural and sustainable materials, the research highlights an alternative to synthetic options. Rigorous experimentation and detailed analysis under identical conditions reveal that both kernels significantly improve heat retention and water production rates. The HSS with date kernels (HSSDK) achieved a daily water productivity of 6.66 kg/m^2^ day, representing an efficiency increase of 10.87%, while the HSS with olive kernels (HSSOK) produced 8.00 kg/m^2^ day, enhancing efficiency by 13.54%. The cost per m^3^ of distilled water for HSSDK is approximately USD 4.65, while HSSOK costs USD 3.89, compared to USD 7.83 for the conventional CHSS system. These results demonstrate that the inclusion of heat storage materials has significantly reduced the cost of water production, with reductions of about 40% for HSSDK and 50% for HSSOK compared to the conventional system. These results are attributed to the high thermal conductivity and specific heat capacities of the kernels, enabling effective heat storage and gradual release. This study demonstrates the potential of agricultural by-products as cost-effective and sustainable solutions for solar water distillation. Further research is recommended to optimize the quantities and configurations of these materials, as well as to explore their integration with other renewable energy systems to enhance overall efficiency and sustainability.

## Introduction

Global water scarcity is escalating due to industrialization, pollution, and rapid population growth, making it imperative to address this issue for the well-being of future generations, as access to clean water is essential for human survival. Solar energy is highly regarded for its simplicity in design, ease of operation, and cost-effectiveness, despite some limitations in productivity. Numerous researchers have focused on enhancing the efficiency of solar stills with single and double slopes, experimenting with various basin configurations^1–4^. Panchal et al.^5^ proposed improvements to solar stills by integrating vacuum tubes and black granite pebbles. Their findings showed that distillate yield from basin materials containing calcium stones exceeded that from black granite gravel and pebbles. Omara et al. (2011)^6^ studied the impact of finned and corrugated solar stills, using 1.5 mm thick iron sheets, in Egyptian conditions, revealing that corrugated and finned solar stills increased productivity by 21% and 40%, respectively, compared to conventional stills when processing 30 L of saltwater. Abderachid and Abdenacer^7^ conducted a comparative analysis of a symmetrical solar still with double effect under varying orientations, Dumka and Mishra^8^ applied Dunkle, Clark, Kumar and Tiwari, Tsilingiris, and a modified version of Spalding’s mass transfer theory to assess the effectiveness of a solar still integrated with a sand bed earth surface, comparing it with a conventional single basin single slope solar still. They utilized the Kumar and Tiwari model to predict yields that closely matched experimental results. Dumka and Mishra conducted a comparative performance analysis of solar stills integrated with soil versus those integrated with earth and a surrounding area coated with transparent polythene and black coal dust powder^9^. According to the review by Srithar and Rajaseenivasan^10^ numerous researchers have conducted comprehensive analyses of methods to enhance heat transfer rates in solar stills^11–13^. These methods include the addition of fins, utilization of energy-storing materials, wicks, incorporation of nanoparticles, cooling with transparent covers, effects of agitation, integration of thermoelectric coolers, multi-effect solar stills, preheating with water heaters, use of photovoltaic thermal collectors, and operation with heat pumps and refrigeration. Selvaraj and Natrajan^14^ xamined various factors influencing solar still performance, such as wind speed, insulation, tilt angle, collector area, temperature difference between water and glass, and solar radiation intensity. In their study on large-scale solar desalination using a seawater aqueduct, Manokar et al.^15^. conducted a comprehensive analysis of both active and passive solar stills combined with PV/T collectors. Olive and Marcoux^16^ proposed a concept where desalination and saltwater occur simultaneously in adjacent pipes, eliminating the need for a clear cover. Arunkumar et al.^17^ investigated the impact of various energy storage materials, such as polyvinyl alcohol (PVA) sponges, pebbles, spherical clay balls (SCBs), and CuO nano-coated absorber plates (CuONCAPs), on the performance of single slope solar stills (SSSS). They found that efficiencies with PVA sponges, pebbles, SCBs, and CuO NCAP were 32%, 44%, 39%, and 53%, respectively, with pebbles showing the highest efficiency. Nocturnal production was also compared. In another study, Kabeel et al.^18^ investigated the effects of suspended nanoscale solid aluminum oxide particles in the basin water of single basin, single slope solar stills with an external condenser. They observed that adding hot air to the basin water increased distillate yield by 108% compared to traditional solar stills^19^ Mishra and Tiwari^20^. conducted a comparative experimental study of single slope solar stills augmented with blackened mild steel chips and single slope earth stills coated with sand mixed with coal powder. Velmurugan et al.^21^ demonstrated increased distillate production in single basin, single slope solar stills by adding fins, wicks, and sponge. Furthermore, Fathy et al.^22^. investigated the effectiveness of double slope stills combined with parabolic solar collectors. Panchal et al.^23^ explored the use of sandstones and marbles as energy storage materials in solar stills, with dripping water used to cool the top cover. Compared to unaugmented stills, those augmented with sandstones and marble pieces showed a 30% and 14% increase in yield, respectively, while also lowering the temperature of the top cover. Abdullah et al.^24^ experimented with tray stills, incorporating modifications such as reflectors and CuO nanoparticles in paint, as well as using paraffin wax mixed with CuO nanoparticles as a phase change material. These enhancements resulted in a 108% increase in fresh water yield and a 51.5% improvement in thermal efficiency for the tray distiller unit. Dubey and Dhananjay^25^ addressed winter challenges of double-slope solar stills, which typically produce less water during winter, by incorporating heat storage materials such as pebbles, iron chips, and black dye. Their modified design increased clean water production by 28.4% and enhanced overall efficiency during winter conditions, offering promising advancements in solar desalination technology. Shoeibi et al.^26^ designed a solar desalination system integrated with phase change materials (PCMs) for district heating, achieving a hot water output valued at 41.94 USD higher than passive solar desalination. Ajdari and Ameri^27^ examined the effects of water flow rates in an Inclined Stepped Solar Still (ISSS) using paraffin wax as PCM combined with CuO and GO nanoparticles, showing that increased flow rates reduced potable water output. However, adding CuO nanoparticles (0.01–0.03%) enhanced water productivity and efficiency by up to 48.12%, while a CuO/GO combination (30:70) increased output by 81.59%. Jobrane et al.^28^ developed a Wick Solar Still (WSS) employing forced convection and enhanced condensation methods, which produced 4 L/m^2^ day, significantly outperforming the conventional solar still’s 2.8 L/m^2^ day.

This study aims to fill this gap by incorporating these natural materials into hemispherical solar stills (HSS). Date kernels (HSSDK) and olive kernels (HSSOK) are used as sensible heat storage units, absorbing solar energy during the day to maintain higher water temperatures for an extended period. The stored heat is gradually released, enabling continued water production even under low sunlight conditions. These materials are not only widely available and cost-effective but also possess unique thermal properties—specifically, high specific heat capacities and good thermal conductivity—that are not commonly exploited in solar distillation systems. This innovative approach not only increases the energy efficiency of the solar still but also boosts the volume of distilled water produced. Our experimental research evaluates how these different insert materials influence the distillation process, focusing on factors such as distilled water volume, system energy efficiency, and overall stability. Through this investigation, we seek to demonstrate that this integrated approach is both practical and effective for water purification. We anticipate significant improvements in both the quantity and efficiency of distilled water production, particularly with the use of date kernel inserts (HSSDK). This research underscores the importance of selecting suitable materials for solar stills and presents a promising solution for enhancing water production in regions facing water scarcity.

## Experimental setup and methodology

**Fig. 1:**
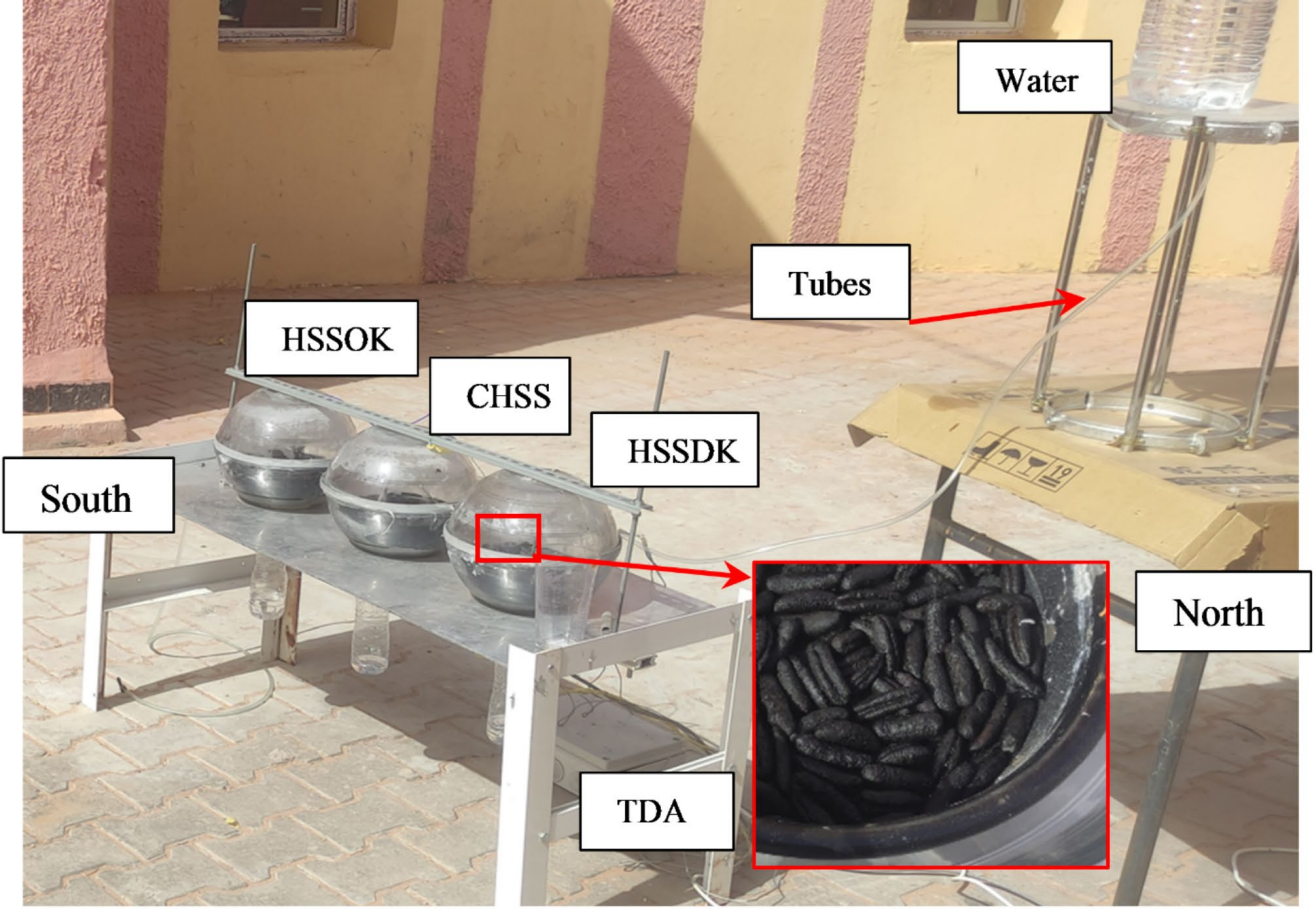
The experimental setup of the proposed Hemispherical Solar Still (HSS) system.

Our experimental setup consists of three identical Hemispherical Solar Stills (HSS), each with a diameter of 30 cm. The first still serves as the control model, maintaining a standard configuration with a black-coated bottom to maximize heat absorption. The second still incorporates date kernels as a heat storage material (HSSDK), while the third utilizes olive kernels (HSSOK). For consistency, 250 g of each kernel type is used, with the kernels uniformly distributed at the base of their respective stills. The kernels are black-coated to enhance heat absorption and remain immersed in the saline water throughout the experiment. These modifications aim to improve the thermal efficiency and water production of the solar stills by leveraging the unique thermal properties of the kernels, such as their high specific heat capacity and thermal conductivity. Figure 1 illustrates the experimental setup of the Hemispherical Solar Still (HSS), comprising a black-painted basin, a transparent hemispherical glass cover, tubes, a collection basin, and various configurations including the Conventional Hemispherical Solar Still (CHSS), the Hemispherical Solar Still with Date Kernels (HSSDK), the Hemispherical Solar Still with Olive Kernels (HSSOK), and the Thermocouple Data Acquisition system (TDA).

A transparent glass cover is positioned above the water surface in each hemispherical solar still to enhance the distillation process. This cover enables sunlight to warm the water while also providing a surface for water vapor to condense into droplets, which then flow down into a hemispherical bowl at the bottom. A 0.5 cm gap between the black basin and the bowl facilitates the collection of purified water droplets. Our experimental setup features a water container connected by 8 mm diameter tubes to three hemispherical solar stills: one conventional, one utilizing date kernels (HSSDK), and one incorporating olive kernels (HSSOK). We used 250 g of date kernels and olive kernels, which were chosen based on prior research and practical considerations. These quantities were optimized based on the available space within the solar still and the typical requirements for effective heat storage without negatively impacting water quality. To maximize heat absorption, the kernels were coated in black to increase their thermal conductivity. Notably, we chose not to dry the kernels, allowing them to remain in their natural state and be immersed in the saline water basin to maintain their properties throughout the distillation process. The black coating enhanced their thermal properties, facilitating better heat retention and gradual release to improve water production rates. To ensure uniformity and aid in reproducibility, we measured the average length of the date kernels to be 1.4 cm, and the olive kernels averaged 0.8 cm. The brackish water is contained in a black-coated aluminum basin, enhancing heat absorption and improving distillation efficiency by raising the water temperature. This setup replicates real-world atmospheric conditions, allowing for precise observation and analysis of the distillation process. To simulate actual conditions, we add 30 g/l of NaCl to the water input. Tests will be conducted at our research facility in URAER, Ghardaia City, over three days from July 5 to July 7, 2024, with operations running from 9 AM to 9 PM. Distillate water will be collected hourly, enabling a thorough examination of water production rates and performance variations over time. A Thermocouple Data Acquisition (TDA) system is connected to a computer for accurate and comprehensive data logging. By closely monitoring and analyzing the distillation process, we aim to gain valuable insights into the effectiveness of date and olive kernels in enhancing distillate water output and overall efficiency.

A thorough error analysis was carried out using Holman modeling approaches in order to guarantee the dependability and robustness of our experimental results in the investigation of hemispherical solar stills employing date and olive kernels as heat storage materials. By using this method, we were able to pinpoint and measure any sources of error in the experimental design, guaranteeing the accuracy and dependability of our results. Each component of the setup was thoroughly inspected, documented, and any factors that may cause mistakes were analyzed. Simultaneously, Table 1 offers a comprehensive overview of the equipment used in the experiment, including their technical specifications, functions, measurement ranges, and uncertainties. The inclusion of multiple instruments, such as a solar radiometer, thermocouples, and a thermometer, underscores the importance of precise data collection in this research. The varying levels of uncertainty and measurement ranges highlight the need for careful consideration of data quality and reliability throughout the experiment. By understanding the capabilities and limitations of these instruments, researchers can improve the accuracy and credibility of their findings. In addition, precise climatic data, obtained from the on-site weather station at URAER Ghardaia, provided essential measurements such as ambient temperature, wind speed, and humidity. This data was critical for creating a realistic context for the experiments, ensuring that the results reflect real-world conditions. The integration of local climatic information further enhances the validity and applicability of our research, allowing us to replicate the actual operating conditions of the solar stills. This comprehensive approach strengthens the overall analysis of the experimental outcomes, particularly with respect to the performance of the stills utilizing date and olive kernels for heat storage.

**Table 1:** Details of the measurement devices employed in the investigation.

Instrument image	Instrument	Quantity measured	Uncertainty %	Range
	Solar-100	Solar radiation intensity	± 2%	0–2000 W m^− 2^
	THERMOCOUPLE	Temperature	± 5%	0–400 °C
	Thermometer DM6802	Temperature	± 5%	-50 to 1300 °C
	Keithley 2010 Industrial Multimeters	Temperatures	± 1%	-328-2501

### The thermal efficiency

Tar stilhe thermal efficiency of a hemispherical soll reflects its ability to convert solar energy into distilled water. This metric is key to understanding how well the still utilizes sunlight for water purification, making it critical in evaluating its effectiveness, especially in regions with limited access to clean water. By optimizing thermal efficiency, researchers and engineers can enhance the design and performance of the still, increasing water production while minimizing energy loss. The thermal efficiency (η) can be determined using the following formula, which factors in the system area (A), solar radiation intensity (I), the amount of distillate produced (m), and the latent heat of vaporization (h_fg_)^29^.

$$\begin{aligned} & \eta _{d} = \frac{{\sum {{\dot{\text{m}}} \times {\text{h}}_{{{\text{fg}}}} } }}{{\text{A}} \times {\text{I}}}{\text{A}} \times 100\quad (I) \\ & {\text{h}}_{{{\text{fg}}}} = 2501.67 - 2.389~ \times T_{w} \\ \end{aligned}$$

An essential component of this research is understanding how a hemispherical solar still’s thermal efficiency relates to its capacity to transform sunlight into clean water. This efficiency measure is essential for assessing how well the still performs in producing clean water, particularly in areas with restricted access to freshwater sources. Reaching high thermal efficiency guarantees these systems’ affordability and sustainability, making them more widely available and useful as solutions to problems with water shortages.

## Results

### Meteorological conditions

**Fig. 2:**
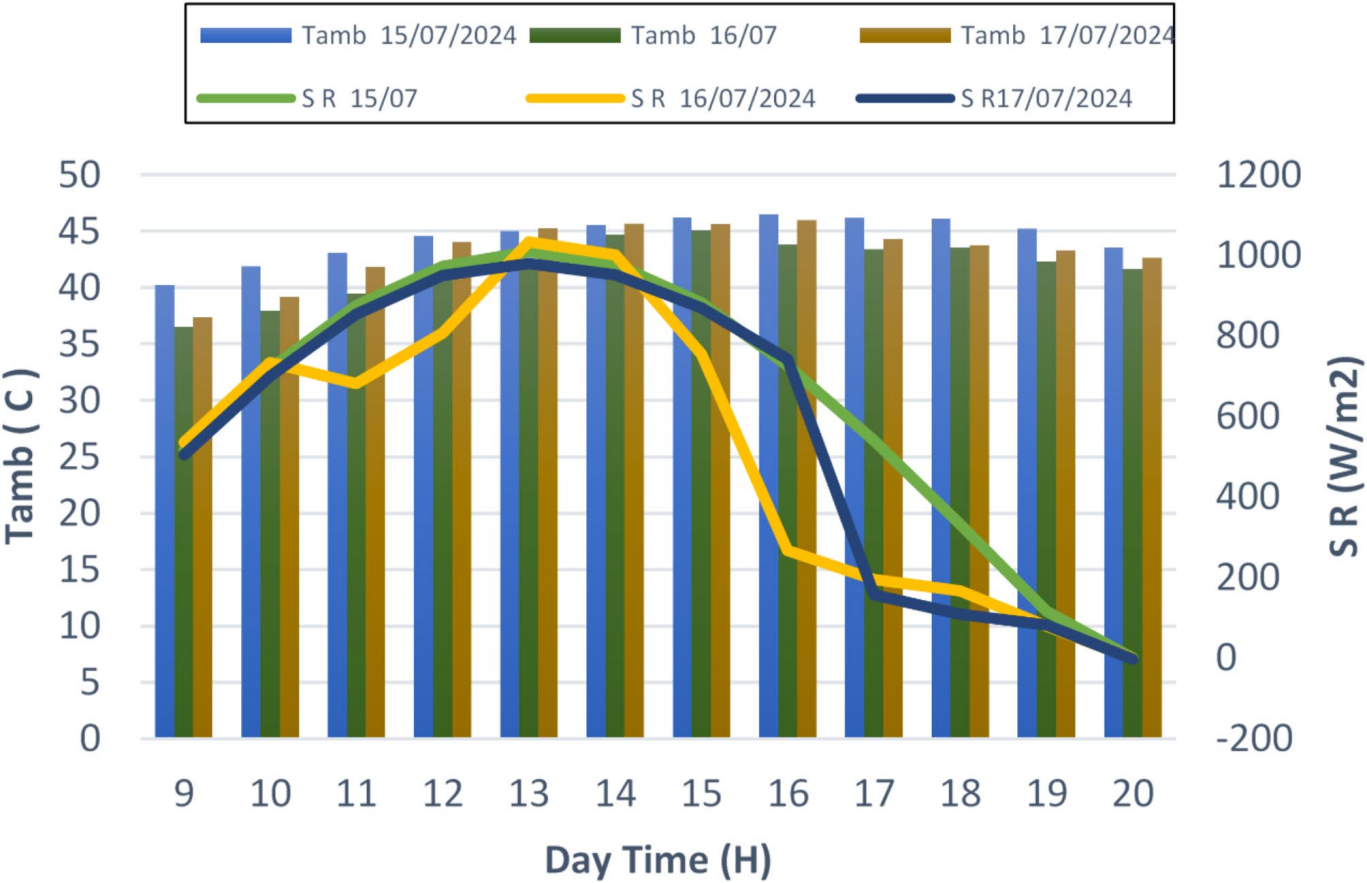
Evolution of ambient temperatures and solar radiation intensity during three days 15/07, 16/07 and 17/07 2024.

It’s critical to comprehend how weather affects distillation procedures, particularly when brackish water is being purified employing solar stills. Temperature, wind speed, humidity, and other meteorological factors have a big impact on the productivity and efficiency of distillation. Humidity affects vapor pressure and air saturation levels, temperature affects the rates of condensation and evaporation, and wind speed affects heat transmission and vapor movement. We can improve distillation systems to generate more clean water from brackish sources by researching how these components interact, which will help address the issue of water shortage in different areas. Figure 2 presents more about these relationships.

#### Solar radiation and ambient temperature

Two key factors influencing the performance of hemispherical solar stills using date and olive kernels as heat storage materials are solar radiation and ambient temperature. Solar radiation serves as the primary energy source for heating the basin water and initiating the evaporation process. At the same time, the ambient temperature plays a critical role in determining the system’s thermal efficiency and the rate of heat loss. Increased solar radiation and higher ambient temperatures enhance the evaporation rate, leading to greater distillation output. Understanding these variations is crucial for optimizing the design and operation of solar stills integrated with date and olive kernels to maximize water production efficiency.

Figure 2 provides an in-depth analysis of the environmental conditions—solar radiation intensity and ambient temperature—observed during the three-day experiment (July 15–17, 2024), evaluating the performance of hemispherical solar stills utilizing date and olive kernels as heat storage materials. The solar radiation intensity (SR) follows a typical diurnal pattern, peaking around midday with values ranging from 300 to 1000 W/m^2^. This underscores the importance of maximizing water evaporation during peak sunlight hours. The incorporation of date and olive kernels in the design plays a key role in storing daytime heat, allowing for sustained high water temperatures even after sunset. This heat retention leads to a significant increase in water production compared to conventional solar still designs. The ambient temperature (T_amb_) shows a predictable fluctuation, starting from lows of around 25 °C at night and potentially rising above 43 °C during the day. These elevated daytime temperatures create a greater temperature differential between the water in the still and the surrounding air, enhancing the evaporation process. The use of date and olive kernels further amplifies this effect by retaining heat within the system, thereby reducing heat loss and extending the evaporation period. By capturing solar energy during peak hours and gradually releasing it throughout the day and night, the date and olive kernels demonstrate their potential to improve the overall efficiency and water production of the system.

#### Humidity and wind speed

This subsection presents the impact of humidity and wind speed on the performance of hemispherical solar stills. Both factors play a crucial role in influencing the evaporation and condensation processes within the system. Higher humidity generally slows evaporation due to the moisture content in the air, while increased wind speed enhances convective heat transfer, aiding in cooling and condensation. In our new work, which focuses on the integration of olive and date kernels as heat storage materials, understanding these environmental factors is essential. The interaction between humidity, wind speed, and the thermal storage capacity of the kernels directly affects the efficiency of water distillation in the solar stills.

**Fig. 3:**
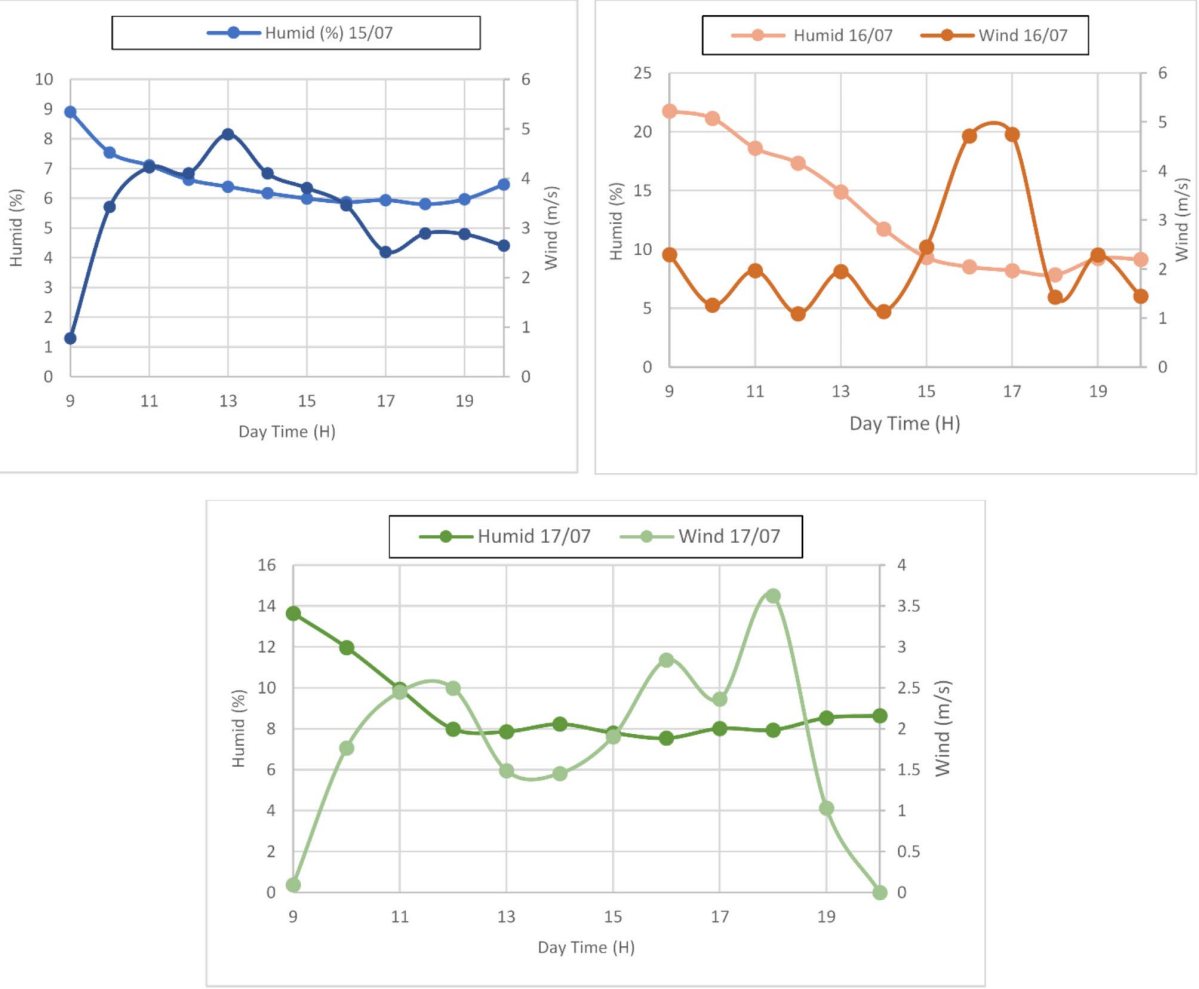
Evolution of wind speed and humidity during three days 15/07 16/07 and 17/07.

Figure 3 illustrates the variations in humidity and wind speed over three days (July 15–17), providing essential data on how these environmental conditions fluctuate. Humidity levels ranged from around 6–22%, with the highest values generally observed in the morning and evening, while wind speeds varied between 1 and 6 m/s, peaking during the midday to afternoon hours. On July 15, for instance, the humidity shows a steady decline from morning to afternoon, while wind speed remains relatively stable, peaking at around 4 m/s. On July 16 and 17, we notice more pronounced fluctuations in wind speed, particularly during the late afternoon, while humidity tends to follow a similar decreasing trend during the day. These variations directly influence the performance of hemispherical solar stills, where higher wind speeds can enhance evaporation, but elevated humidity may slow the condensation process. Relating this to our work on Hemispherical Solar Stills Using Date and Olive Kernels as Heat Storage Materials, the kernels’ heat storage capability plays a critical role in compensating for such environmental variability. As these kernels store heat during peak sunlight hours and release it gradually when solar radiation diminishes, they ensure a more consistent thermal environment inside the still. This reduces the dependency on favorable external conditions like lower humidity or optimal wind speed, allowing for improved water production even under less ideal weather patterns. Thus, integrating date and olive kernels enhances the efficiency of the stills by stabilizing their internal temperature, making them more effective in diverse climatic conditions.

#### Glass cover temperature and solar radiation

In this subsection, we examine how the temperature of brackish water changes in relation to sunlight intensity, a key factor in optimizing the performance of our hemispherical solar stills using date kernels (HSSDK) and olive kernels (HSSOK) as heat storage materials. As sunlight intensity increases, the water inside the still is heated more efficiently, which accelerates the evaporation process. The stored heat in the kernels plays a crucial role by retaining thermal energy even after peak sunlight hours, allowing for prolonged water heating and evaporation. By analyzing how sunlight impacts water temperature, we can fine-tune our solar stills with date and olive kernels to improve efficiency, ultimately producing more clean water, particularly for water-scarce regions.

**Fig. 4:**
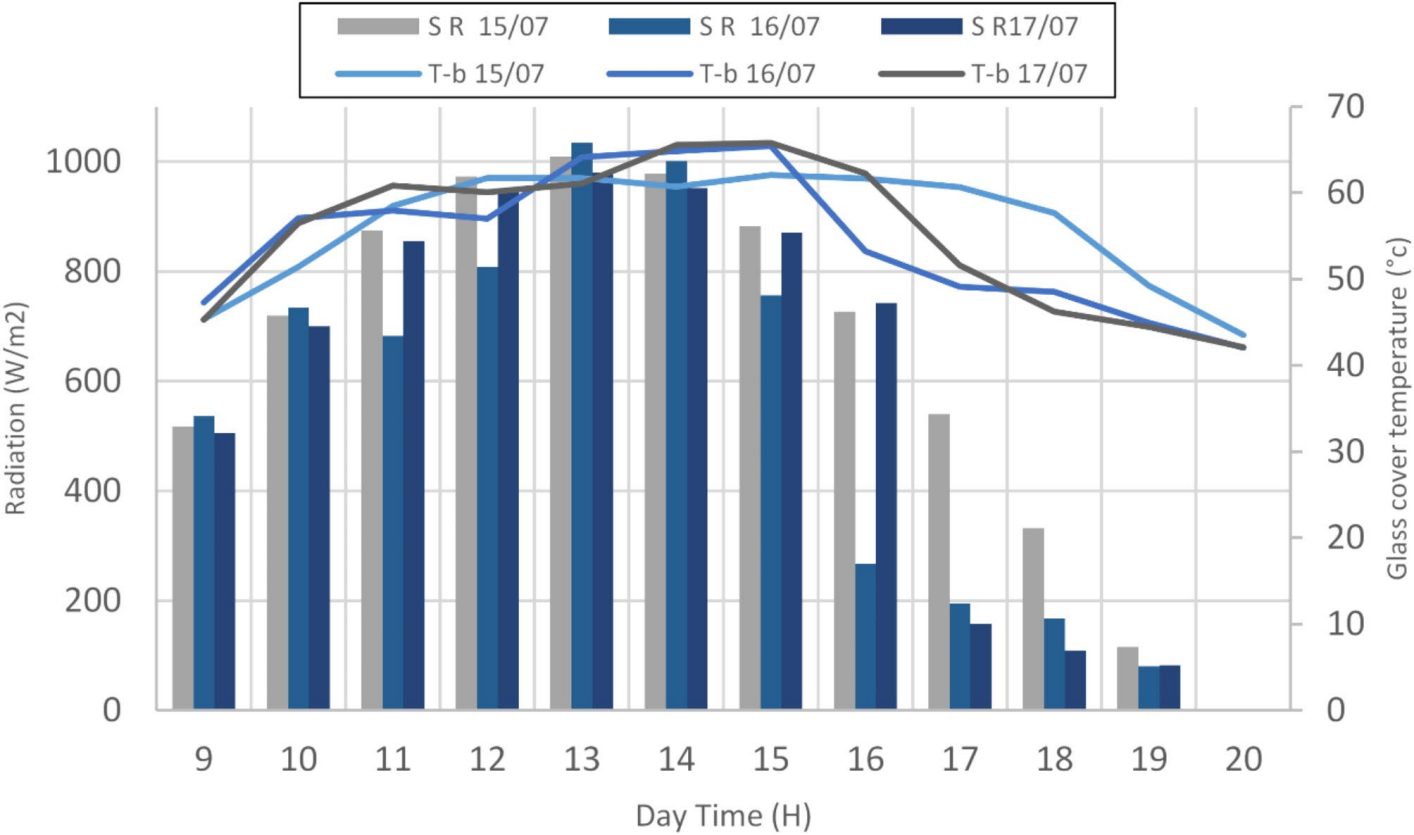
Variation of surface glass cover temperature in the Conventional hemispherical solar stills (CHSS in relation to solar radiation intensity over three days, from July 15th to 17th.

Figure 4 illustrates the relationship between solar radiation intensity (SR) and glass cover temperature (T-b) in hemispherical solar stills equipped with date and olive kernels as heat storage materials, recorded over three days from July 15th to July 17th. The solar radiation intensity peaked at around 900 W/m^2^ on July 15th and 16th, resulting in T-b values reaching approximately 65 °C on July 17th. Notably, minimum T-b values were recorded during nighttime, typically falling below 20 °C. This data underscores the critical connection between solar energy input and glass cover temperature, which is essential for optimizing the performance of solar water distillation systems. The inclusion of date and olive kernels as heat storage materials enhances the thermal efficiency of the stills, allowing for improved heat retention and extended water distillation even after solar radiation diminishes. By leveraging the thermal properties of these natural materials, our research aims to demonstrate significant improvements in water production rates and overall system efficiency. The findings from this analysis provide valuable insights into how optimized temperature management can facilitate better water purification solutions, particularly in regions facing water scarcity.

## Effects of storage materials

### Basin and brackish water temperature evolution

In this subsection, we examine the interplay between basin surface temperatures, humidity, and wind speed within our hemispherical solar stills utilizing date and olive kernels as heat storage materials. By analyzing how these environmental factors interact, we gain critical insights into the thermal dynamics of our experimental setup. Basin surface temperatures are significantly influenced by ambient humidity and wind speed, both of which are essential for heat transfer processes within the solar still. Humidity affects the rate of evaporation by altering the air’s moisture content, while wind speed influences convective heat transfer, enhancing or diminishing the cooling effect on the surface. Understanding these interactions is vital for optimizing the performance of our distillation apparatus. By leveraging these environmental conditions, we aim to improve the efficiency and water production yield of our solar stills. This comprehensive analysis will provide a clearer understanding of how to refine the system’s design and operation, ultimately leading to enhanced performance in real-world applications.

**Fig. 5:**
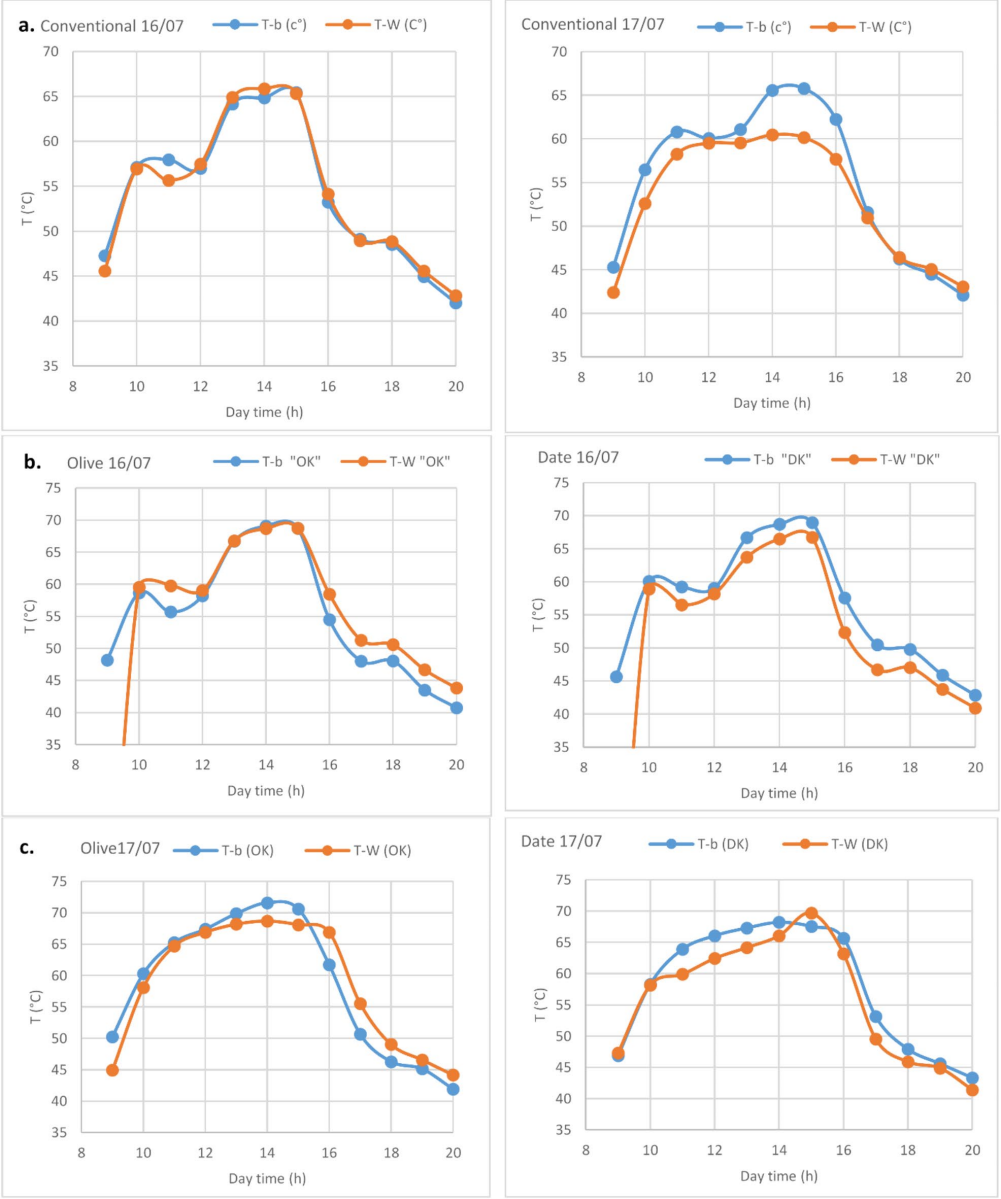
Basin temperature and brackish water evolution as function of daytime for conventional solar still, solar still with olives kernels and solar till with dates kernels during July 16th and 17th July.

Figure 5 presents a detailed comparison of the thermal performance of hemispherical solar stills using olive and date kernels as heat storage materials. Figure 5.a shows the daily variations in basin temperature (T-b) and water temperature (T-w) for a conventional still. A strong positive correlation is evident between T-b and T-w, highlighting effective heat transfer; however, the water temperature remains slightly lower than the basin temperature, indicating room for improvement in heat transfer efficiency. Figure 5b, focused on July 16th, compares the performance of stills enhanced with olive and date kernels. The olive kernel still consistently achieves higher basin and water temperatures throughout the day, demonstrating its superior heat retention properties compared to the date kernel still. This suggests that olive kernels are more effective in capturing and storing solar energy, contributing to improved water heating and overall system performance. Figure 5c, focused on July 17th, continues this comparison, showing that the olive kernel still consistently outperforms the date kernel still. The higher basin and water temperatures achieved by the olive kernel still reaffirm its potential as a highly effective heat storage material, leading to enhanced evaporation rates and increased distillate yield. The inclusion of kernels, particularly olive kernels, in our hemispherical solar still design is a crucial factor in optimizing system performance. Acting as natural heat storage materials, kernels absorb excess solar energy during peak hours and release it gradually, maintaining higher water temperatures even when solar radiation decreases. This extended heat retention improves the distillation efficiency of the system. In our new work, the use of olive kernels has proven to significantly enhance the effectiveness of solar water distillation systems. Further research will help refine the application of kernels and explore additional heat storage options to further improve performance.

### Brackish water and radiation intensity

In this subsection, we examine the relationship between radiation intensity and the temperature of brackish water in the context of our enhanced hemispherical solar stills utilizing olive and date kernels as heat storage materials. The intensity of solar radiation is a key factor influencing the heating of the water, which directly impacts the evaporation and condensation processes. By understanding how varying radiation levels affect the water temperature, we can optimize the thermal storage capabilities of the kernels and improve the overall efficiency of the solar still. This analysis is crucial for refining the use of natural heat storage materials in solar distillation systems and maximizing water production.

**Fig. 6:**
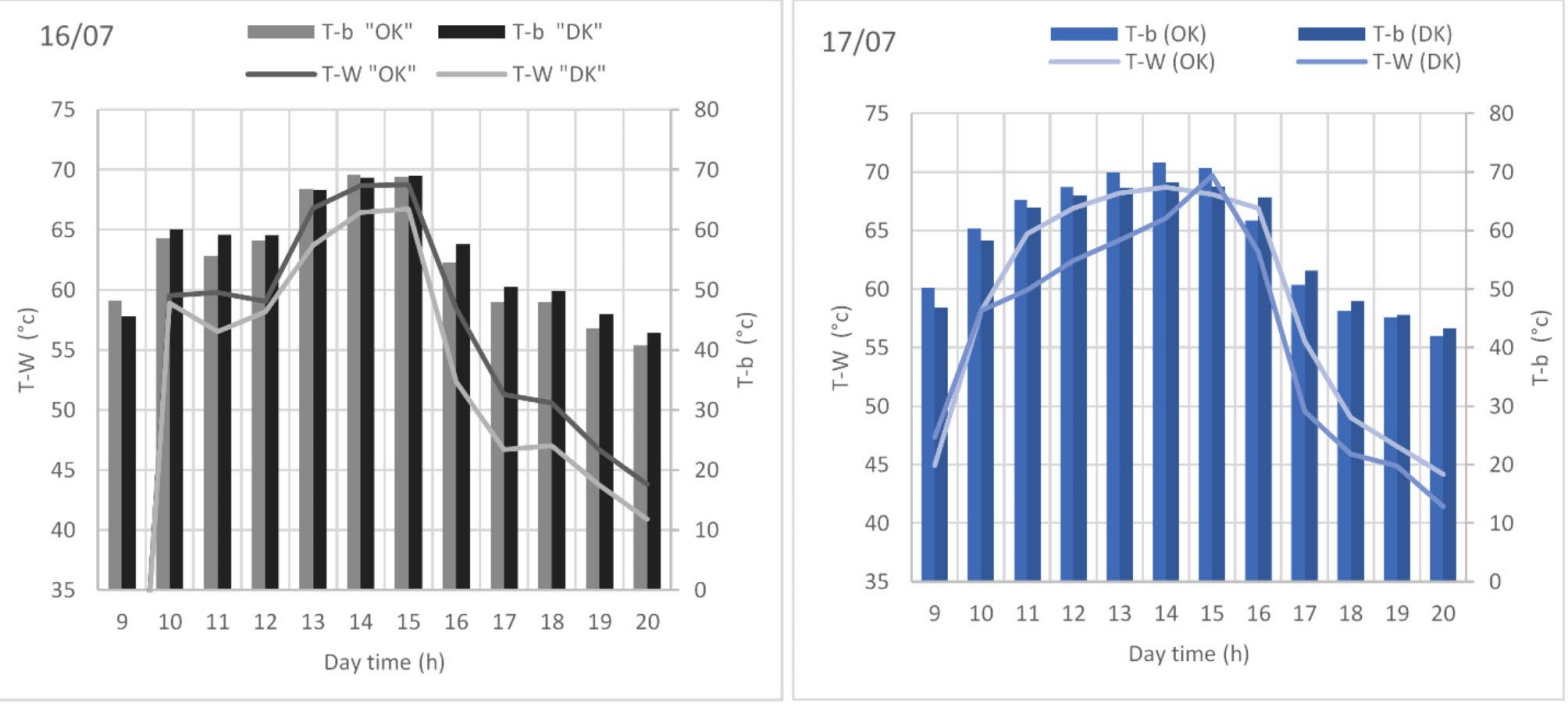
Evolution of brackish water (T-W) and basin temperatures (T-b) for both HSSOK (OK) and HSSDK (DK) as function of daytime during 16/07 and 17/07.

In Fig. 6, we analyze the evolution of brackish water temperature (T-W) and basin temperature (T-b) for both hemispherical solar stills with olive kernels (HSSOK) and date kernels (HSSDK) over July 16th and 17th. The maximum T-W recorded for HSSOK on July 16th reached around 67 °C at 14:00, while the corresponding T-W for HSSDK was approximately 62 °C. Similarly, the basin temperature (T-b) for HSSOK peaked at around 75 °C, whereas for HSSDK, it only reached about 70 °C. On July 17th, a similar trend was observed, with HSSOK again achieving higher temperatures—T-W peaked at 66 °C for HSSOK and 61 °C for HSSDK, while T-b hit 74 °C and 69 °C, respectively. These results demonstrate that olive kernels, with their superior thermal capacity, allow for better heat retention and transfer within the system. The higher temperatures sustained by HSSOK directly contribute to more effective evaporation of brackish water, thereby improving the efficiency of the distillation process.

**Fig. 7:**
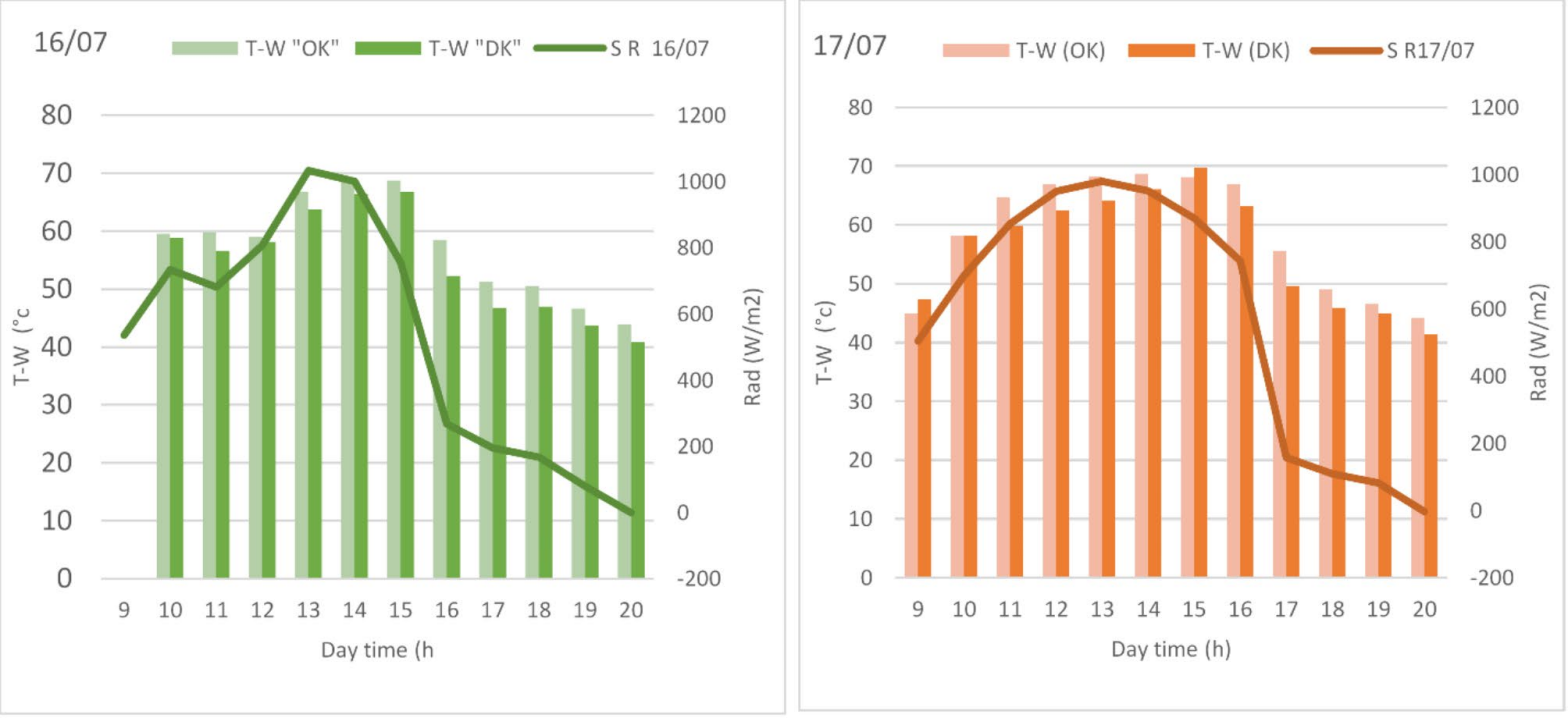
Evolution of brackish water (T-W) as function of daytime solar radiation (Rad) for both HSSOK (OK) and HSSDK (DK) during 16/07 and 17/07.

In Fig. 7, the relationship between T-W and solar radiation intensity (Rad) for both systems is highlighted during the same period. Solar radiation reached its maximum at around 950 W/m^2^ on July 16th and 17th. During peak radiation at 14:00, HSSOK consistently exhibited higher T-W values—approximately 67 °C on July 16th and 66 °C on July 17th—compared to HSSDK, which only achieved 62 °C and 61 °C, respectively. The heat retention by the olive kernels is particularly notable in the late afternoon, as the T-W in HSSOK remains higher even as solar radiation declines to below 200 W/m^2^. By contrast, HSSDK shows a sharper decline in temperature, indicating lower heat retention efficiency. The key factor behind this performance lies in the thermal properties of the kernels. Olive kernels, with their higher density and specific heat capacity compared to date kernels, store more solar energy and release it gradually over time, providing a more stable and extended heat supply. This feature is crucial for solar stills, as it ensures that water temperatures remain elevated for longer periods, even during times of reduced solar radiation, thus maximizing evaporation rates and increasing freshwater yield. In our experiments, HSSOK showed a 5–6 °C temperature advantage over HSSDK during peak solar hours, leading to a significant boost in water production. These findings validate the use of olive kernels as a more efficient heat storage medium in hemispherical solar stills compared to date kernels. The increased thermal performance enhances the overall distillation efficiency by promoting more consistent and prolonged evaporation, making olive kernels a promising material for optimizing solar water desalination technologies.

## Distillation efficiency analysis

### Distillation yield analysis

This subsection presents the analysis of distillation efficiency in hemispherical solar stills. Distillation efficiency is a critical measure of the system’s ability to convert brackish water into distilled water by utilizing solar energy. Factors such as solar radiation, basin temperature, heat storage materials, and environmental conditions (humidity and wind speed) all influence the distillation efficiency. In our new work, where olive and date kernels are used as heat storage materials, the efficiency analysis helps determine how well these materials enhance the system’s ability to store and release heat, ultimately improving water yield and overall performance of the solar still.

**Fig. 8:**
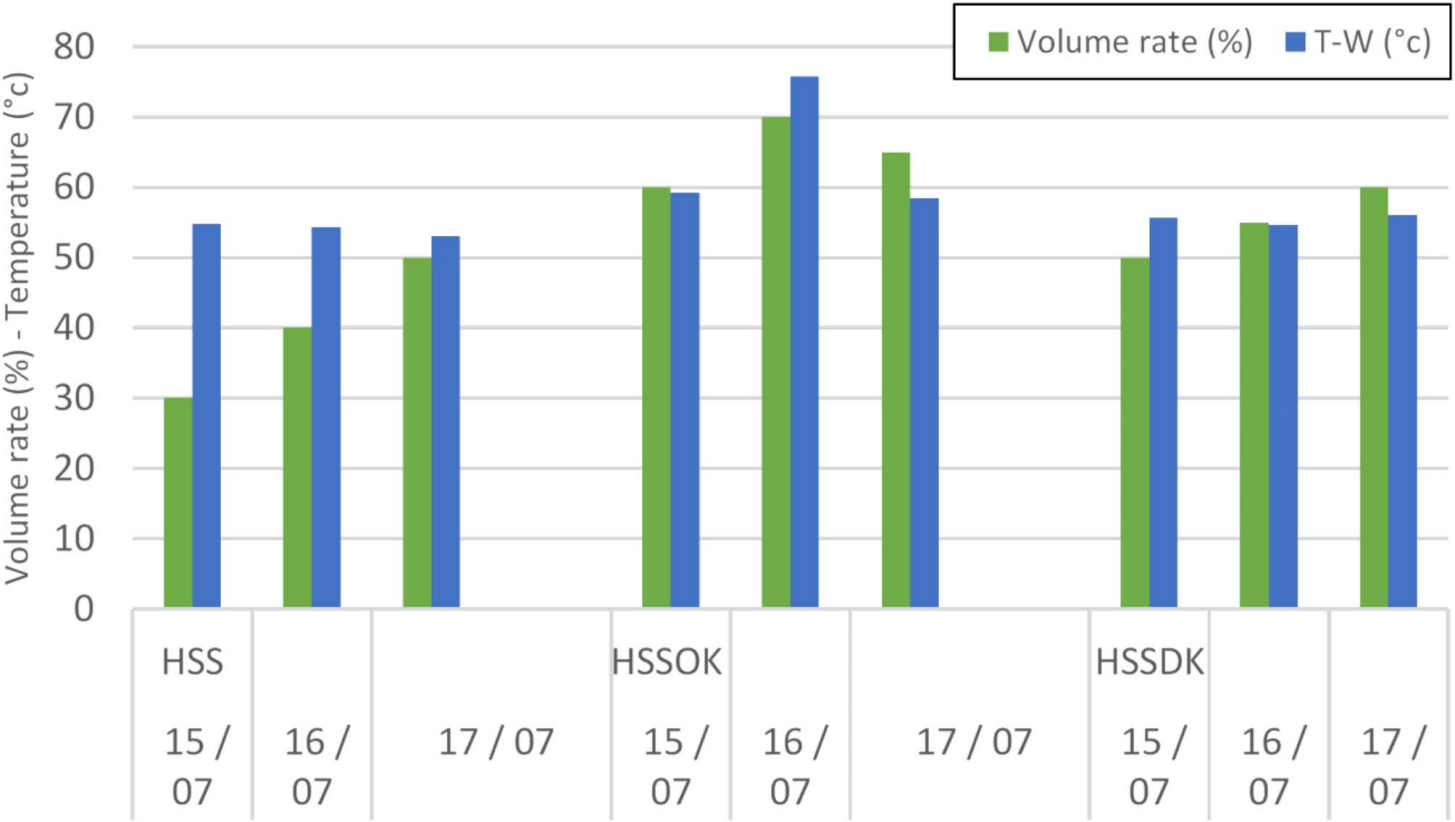
The distillate collected at 21 PM for the three days 15, 16 and 17/07 for all of HSS, HSSOK and HSSDK.

Figure 8 shows that the hemispherical solar still enhanced with olive kernels (HSSOK) consistently produces more distillate compared to the date kernel system (HSSDK) and the conventional still (HSS). On July 16th, for instance, HSSOK achieves a distillate rate of approximately 70% with a water temperature of around 75 °C, while HSSDK produces about 60% at 70 °C. The higher distillate production in the HSSOK can be attributed to the superior heat storage capacity of olive kernels, which allows them to absorb and retain heat more effectively during periods of peak solar radiation. Olive kernels have higher specific heat capacity and thermal conductivity compared to date kernels, which means they store more energy during the day and release it gradually, keeping the water temperature elevated for longer periods. This sustained heat release prolongs evaporation, even during late afternoon hours when solar radiation declines, resulting in a higher distillate yield by the end of the day. The conventional HSS, without any thermal storage material, shows lower distillate rates (40–55%), confirming the importance of incorporating effective heat storage materials like olive kernels to improve the overall efficiency of solar stills.

### Distillation performance

In this subsection, we explore the thermal efficiency of three configurations of hemispherical solar stills: the conventional still (HSS), the still with olive kernels as heat storage (HSSOK), and the still with date kernels as heat storage (HSSDK). Thermal efficiency is a key indicator of a solar still’s performance, reflecting its ability to convert absorbed solar energy into usable thermal energy for water distillation. The use of olive and date kernels as heat storage materials is intended to enhance the stills’ efficiency by retaining heat longer, allowing for sustained water evaporation even when solar radiation decreases. By comparing these configurations, we aim to identify the most effective design for optimizing energy use, improving distillate yield, and advancing sustainable water purification methods. This approach emphasizes the potential of natural heat storage materials in improving solar distillation efficiency for practical, environmentally friendly applications.

**Fig. 9:**
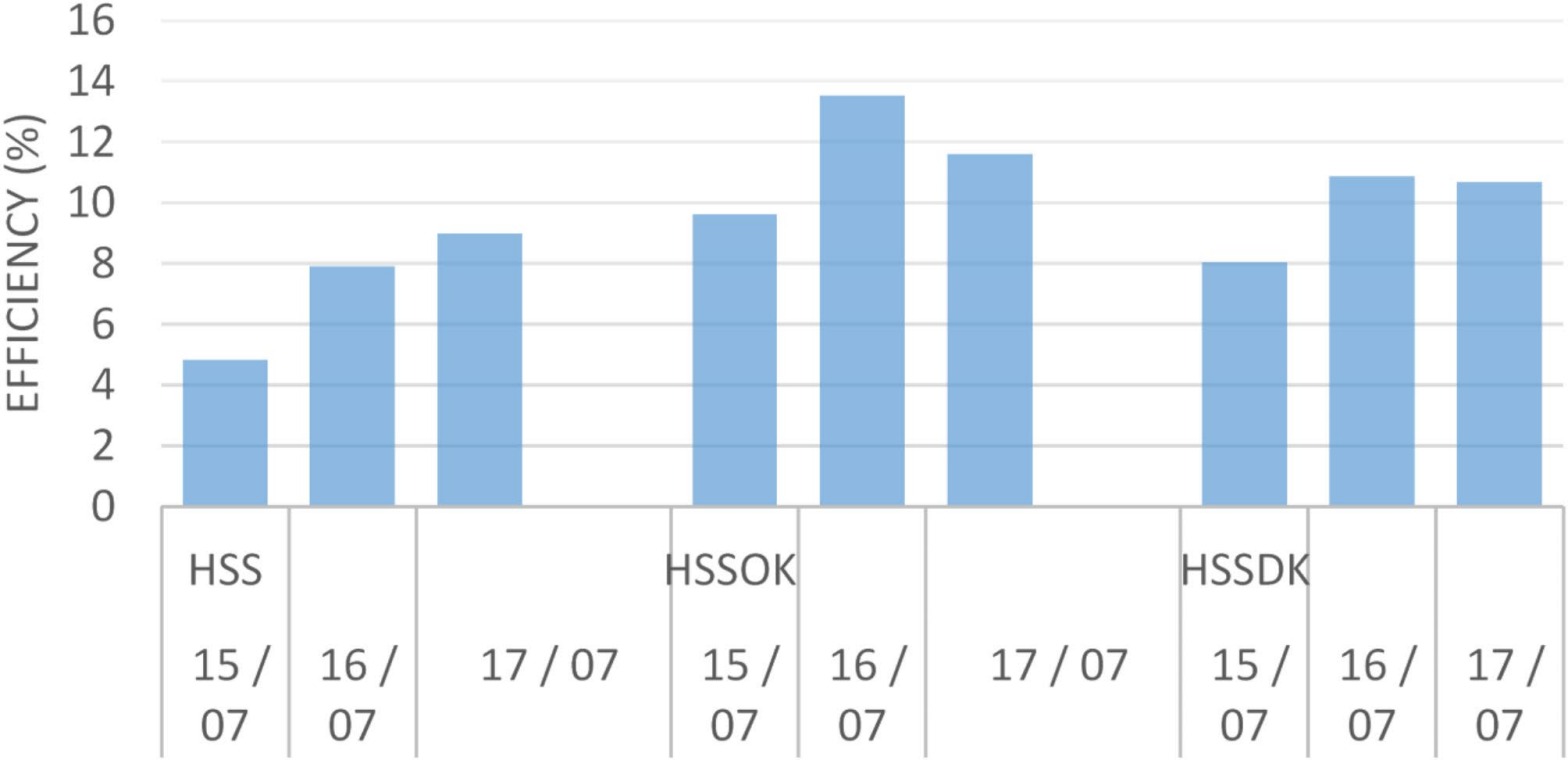
Thermal efficiency of three configurations in two days 06/07 and 07/07.

Figure 9 illustrates the thermal efficiency of three configurations: the conventional hemispherical solar still (HSS), the solar still with olive kernels (HSSOK), and the solar still with date kernels (HSSDK) over two days (15/07 and 16/07). HSSOK consistently exhibits higher thermal efficiency compared to both HSS and HSSDK. This increased efficiency can be attributed to the superior thermal properties of olive kernels. Olive kernels have a higher specific heat capacity (approximately 2.5 J/g°C) and thermal conductivity (0.35 W/m°C), allowing them to absorb and store more heat during sunlight hours. This stored heat is released gradually, sustaining a higher water temperature over time. This extended heat retention promotes continuous evaporation even when solar radiation decreases, which is crucial for maintaining the distillation process during periods of low sunlight. In comparison, date kernels, which have a lower specific heat capacity (approximately 1.8 J/g°C) and thermal conductivity (0.30 W/m°C), release stored heat more quickly. This faster release leads to quicker cooling and less sustained evaporation, resulting in lower water temperature retention and reduced efficiency in the distillation process. The efficient heat retention of olive kernels creates a more stable thermal environment in the solar still, enhancing the stability and consistency of the water production. This stability allows for better management of thermal losses, making HSSOK the most effective configuration among the three tested setups. The use of olive kernels as heat storage material is particularly beneficial in maintaining optimal operating temperatures, which improves the overall distillation efficiency and water output.

### Comparison with previous research

This subsection presents a comparative analysis between our hemispherical solar still using olive and date kernels as heat storage materials and previous solar still designs. By examining the impact of different additives and modifications on daily water output and efficiency, we position our findings within the broader scope of solar distillation advancements (see Table 3). This comparison showcases the improvements made through our use of natural kernel materials, underscoring the effectiveness of olive and date kernels in enhancing distillation performance. The table below summarizes findings from various studies on solar still designs, including enhancements like wicks, internal reflectors, external condensers, and additives such as nanoparticles. For each design, we list the daily water output in kilograms per square meter per day (kg/m^2^ day) and the percentage increase in efficiency relative to a baseline design, helping to highlight the distinct advantages of kernel-based modifications for sustainable water production.

**Table 2:** Comparison of our work and previous studies on solar still designs, modifications, additives, productivity, and rate of increase.

Design of SS	Ref.	Modification/Additives	Productivity (kg/m^2^.day)	Rate increase (%)
Single slope solar distiller	Elango et al.^30^	-0.1 wt% SnO_2_ NF	0.805	18.62
		-0.1 wt% Al_2_O_3_ NF	0.935	29.95
		-0.1 wt% ZnO NF	0.750	12.67
Tubular solar still	Kabeel et al.^31^	Wicks and v-corrugated basin	6.01	6.010
Trays solar distiller	Abdullah et al.^24^	-Internal reflectors	3.45	57.00
		-Reflectors + CuO nano black paint	4.10	70.70
		-Reflectors + CuO nano black paint + CuO nano mixed PCM	5.00	108.0
Single slope solar distiller	Johnstone Madhlopa^32^	External condenser	6.08	62.01
Tubular solar still	Elshamy and El-said^33^	Hemi-circular v-corrugated basin	4.30	26.47
Hemispherical solar distiller with kernels	Present work	Hemispherical solar stills with olive and date kernels (HSSOK & HSSDK)	8 HSSOK	13.54
			6.66 HSSDK	10.87

Table 2 provides a comparative analysis of various solar still designs and their enhancements, illustrating the influence of modifications and additives on productivity and efficiency rates. For single slope solar distillers, nanofluids like Al_2_O_3_, SnO_2_, and ZnO lead to substantial productivity increases, with Al_2_O_3_ achieving the highest productivity at 0.935 kg/m^2^/day, marking a 29.95% improvement. Tubular solar stills equipped with wicks and v-corrugated basins reached a productivity of 6.01 kg/m^2^/day, showing a notable increase. Tray solar distillers demonstrated even greater efficiency gains with reflective modifications: internal reflectors alone provided a 57% increase, while combining reflectors, CuO nano black paint, and PCM further boosted productivity to 5.00 kg/m^2^/day with a 108% increase. Additionally, single slope solar stills with external condensers reached 6.08 kg/m^2^/day, reflecting a 62.01% increase in efficiency. In our current work on hemispherical solar stills incorporating olive and date kernels (HSSOK and HSSDK), the productivity rates are among the highest, with HSSOK achieving 8 kg/m^2^/day (13.54% increase) and HSSDK yielding 6.66 kg/m^2^/day (10.87% increase). This indicates that natural materials like olive and date kernels not only enhance the thermal storage capacity but also boost distillation efficiency significantly. These findings suggest that organic additives can offer a sustainable, high-performance alternative to synthetic materials for optimizing solar still productivity.

### Solar still cost

The economic assessment of hemispherical solar stills incorporating olive and date kernels (HSSOK and HSSDK) is essential to determine their practicality for real-world applications. This subsection presents a detailed cost analysis of the materials, fabrication, and operational expenses associated with the HSSOK and HSSDK systems. Particular attention is given to the affordability and availability of olive and date kernels as natural, low-cost heat storage materials in regions where these resources are abundant. The analysis evaluates the trade-off between the additional costs of integrating thermal storage materials and the enhanced distillation efficiency achieved. Comparisons are drawn between the cost-effectiveness of the HSSOK and HSSDK configurations and the conventional hemispherical solar still (HSS). This discussion provides insights into the scalability and financial feasibility of implementing these systems for sustainable water production in cost-sensitive regions. The cost of each configuration as well as the daily amount of produced water (L/m^2/^day) for five years are presented in Table 3.

**Table 3:** Different cost of HSSDk, HSSOK and CHSS.

Cost (DZD)	HSSDK	HSSOK	CHSS
Manufacturing cost (DZD)	2000	2000	2000
Tubes, Basin, glass cover	1200	1200	1200
Support	100	100	100
Heat Storage Materials	100	150	0
Maintenance cost (DZD)	30	30	30
Total cost of the prototype	3430	3480	3430

Table 3 compares the costs of three hemispherical solar still configurations: HSSDK (with date kernels), HSSOK (with olive kernels), and CHSS (conventional without heat storage materials), with costs converted from DZD to USD at a rate of 1 USD = 133.894 DZD (the exchanges rates of Dec 23th 2024). The manufacturing costs, including tubes, basin, hemispherical glass (USD 8.96) and support (USD 0.75), are consistent across all systems. The inclusion of heat storage materials slightly increases the total cost, with HSSDK incurring USD 0.75 for date kernels and HSSOK USD 1.12 for olive kernels, while CHSS has no additional cost for heat storage. Maintenance costs are uniform across all configurations at USD 0.22, leading to total costs of USD 25.62 for HSSDK and CHSS, and USD 25.99 for HSSOK. Using the relation.

$${\text{Cost per m}}^{3} = {\text{Total Cost /Annual Water Production}}$$

The cost per m^3^ of distilled water is approximately USD 4.65 for HSSDK, USD 3.89 for HSSOK, and USD 7.83 for CHSS. These estimations are made for 1 m^2^ of the water basin, as our prototype has a small area of 0.023 m^2^. Notably, the integration of heat storage materials has led to a reduction in the cost of water production compared to the conventional CHSS system. The cost per m^3^ for HSSDK and HSSOK is reduced by approximately 40% and 50%, respectively, showcasing the effectiveness of incorporating heat storage in reducing operational costs, while offering potential performance benefits. HSSDK remains cost-effective, balancing affordability and efficiency, making it a competitive option for solar distillation systems.

## Conclusion

In conclusion, our study highlights the substantial benefits of using olive and date kernels as thermal storage materials in hemispherical solar stills, significantly improving water distillation performance. Through systematic testing and analysis, we demonstrated that these natural, organic additives enhance both heat retention and distillation efficiency, resulting in higher water output compared to conventional designs. Our key findings include:

- The use of heat storage materials, such as date and olive kernels, significantly influences the performance of solar stills. These materials enhance heat retention and transfer, leading to higher water temperatures and improved evaporation rates.
- Olive kernels (HSSOK) achieved higher brackish water temperatures (T-W) compared to date kernels (HSSDK) during the testing period, demonstrating superior heat retention and transfer capabilities, which improved evaporation efficiency.
- The olive kernels’ higher density and specific heat capacity allowed them to store more solar energy and maintain elevated water temperatures even when solar radiation decreased. This resulted in a temperature advantage of 5–6 °C for HSSOK during peak solar hours, significantly boosting water production and overall distillation efficiency.
- Olive kernels proved to be particularly effective, achieving a daily productivity of 8 kg/m^2^ day, with an efficiency increase of 13.54%, while date kernels reached 6.66 kg/m^2^ day, corresponding to a 10.87% increase in efficiency.
- The enhanced thermal properties of olive kernels contributed to their superior performance, allowing for more sustained heat release during the distillation cycle.
- These results emphasize the viability of natural materials as cost-effective and eco-friendly alternatives to synthetic additives in solar still designs.

This work underscores the potential of incorporating bio-based thermal storage materials in solar distillation technology, offering a sustainable solution to water scarcity issues, particularly in arid and semi-arid regions. Continued exploration of natural materials can further enhance the performance, efficiency, and environmental sustainability of solar water purification systems, contributing to accessible clean water solutions worldwide.

## Data Availability

Data available on request from the authors. The data that support the findings of this study are availablefrom the corresponding author, [Dr. Takele Ferede Agajie], upon reasonable request.
